# Supplementary material for: ICG-001, an Inhibitor of the β-Catenin and cAMP Response Element-Binding Protein Dependent Gene Transcription, Decreases Proliferation but Enhances Migration of Osteosarcoma Cells
Source: Pharmaceuticals (Basel). 2021 May 1;14(5):421. doi: 10.3390/ph14050421 (PMC8147379; doi:10.3390/ph14050421)
Supplement: Supplementary file 1 [file pharmaceuticals-14-00421-s001.zip › Supplementary figures and legends.pdf]

Supplementary figures

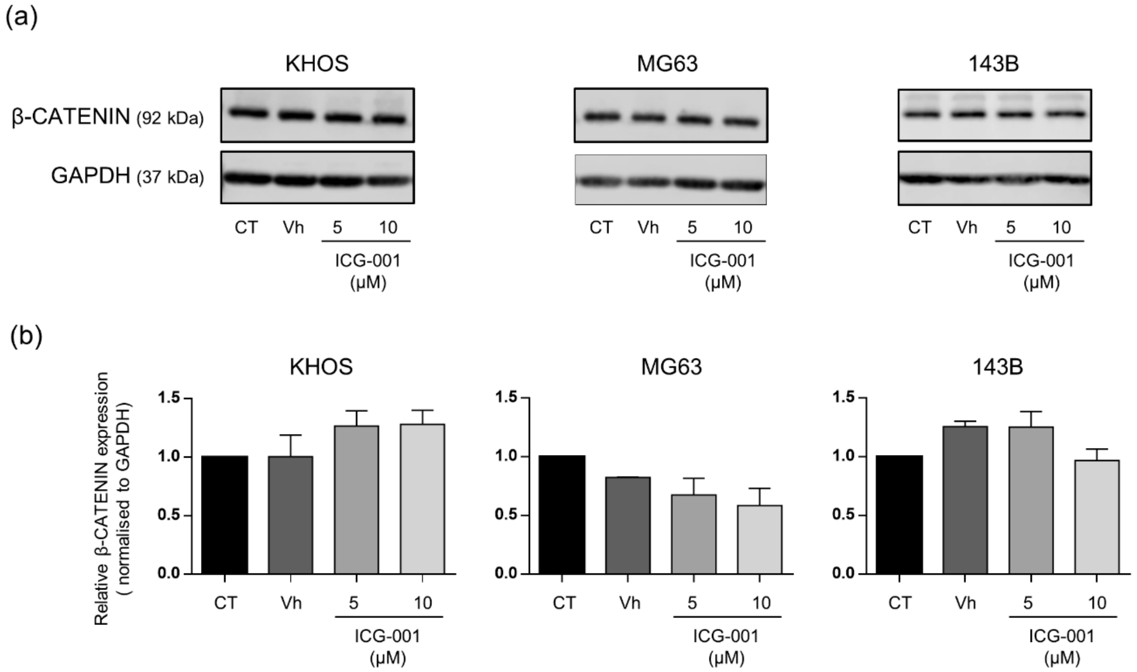

Supplementary figure S1: ICG-001 does not decrease β-CATENIN protein level in KHOS, MG63 and 143B osteosarcoma cells. (a) β-CATENIN protein level evaluated by Western Blot in osteosarcoma cell lines treated with Vehicle (Vh) or ICG-001 (5-10μM) during 24 hours (one representative experiment of two independent experiments). (b) Relative β-catenin protein quantification, normalised with GAPDH protein expression. Bars indicate means ±SD of two independent experiments.

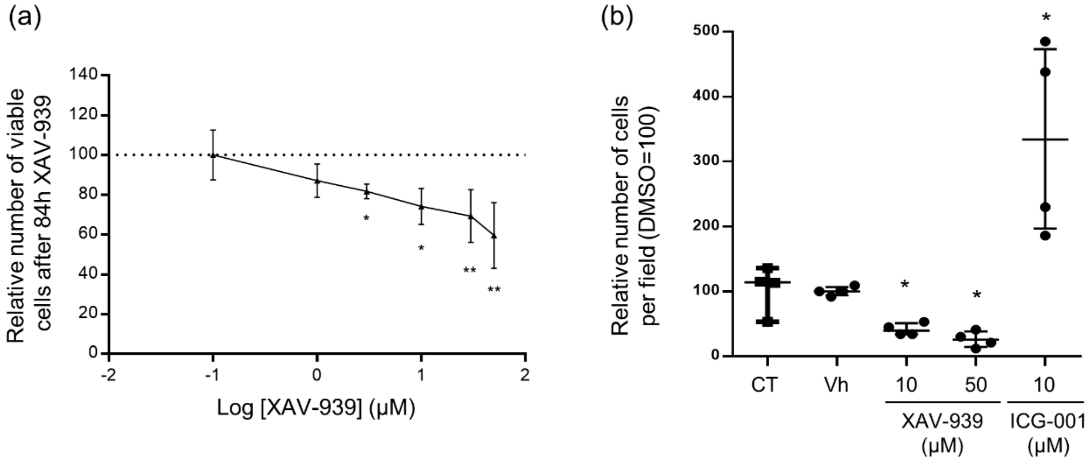

Supplementary figure S2: XAV-939 decreases viability and migration of KHOS osteosarcoma cells. (a) Relative number of viable cells analysed in cells treated with XAV-939 or vehicle (Vh) from 1 to 50 μM for 48 hours. At the end points, cells were stained with crystal violet and the curves represent the means ±SD relativized with the value of the vehicle (DMSO), of 3 independent experiments, each performed in triplicate. The dashed line represents the vehicle value set to 100. *, $p<0.05$; **, $p<0.01$ vs vehicle. (b) Migrated KHOS cells counting after 24h of XAV-939 (10μM or 50 μM), ICG-001 (10μM) or vehicle (Vh) pre-treatment and seeding in Boyden Chamber for 8h. Bars represent means ±SD (N=3, each performed in duplicate). *, $p<0.05$ vs vehicle.
