# Supplementary figures and images for: ICG-001, an Inhibitor of the β-Catenin and cAMP Response Element-Binding Protein Dependent Gene Transcription, Decreases Proliferation but Enhances Migration of Osteosarcoma Cells
Source: Pharmaceuticals (Basel). 2021 May 1;14(5):421. doi: 10.3390/ph14050421 (PMC8147379; doi:10.3390/ph14050421)

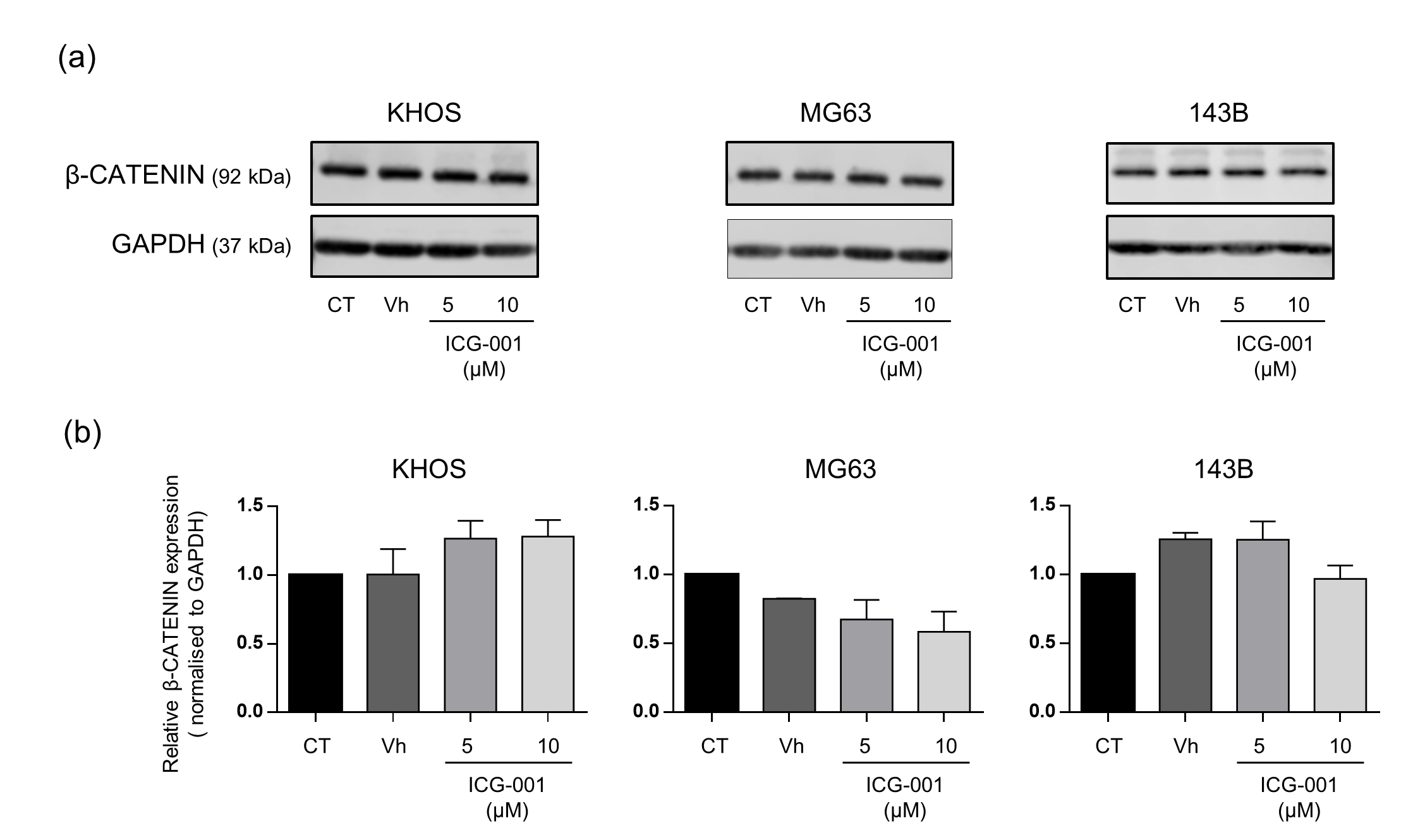

Supplement: Supplementary file 1 [file pharmaceuticals-14-00421-s001.zip › Supplementary figures/Supplementary figure S1.tif]

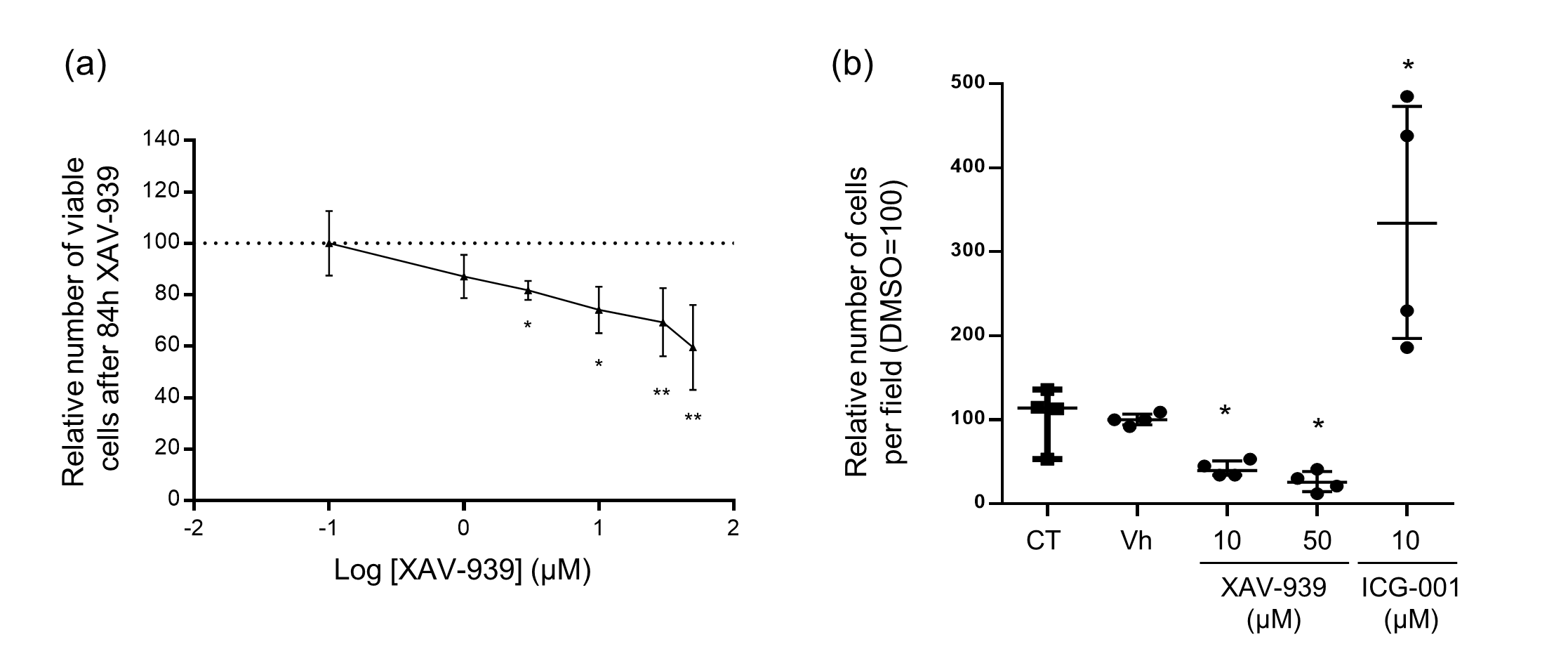

Supplement: Supplementary file 1 [file pharmaceuticals-14-00421-s001.zip › Supplementary figures/Supplementary figure S2.tif]
